# Intraosseous Lipoma Mimicking Periapical Lesion: A Rare Presentation

**DOI:** 10.1155/2022/5462352

**Published:** 2022-11-02

**Authors:** Abhishek Bannerjee, Snehashish Ghosh, Moumalini Das, Patrick Wang, Saranya Ramsridhar, Shankargouda Patil

**Affiliations:** ^1^Department of Oral Pathology, Avadh Dental College and Hospital, Jamshedpur, India; ^2^Department of Oral Pathology, College of Medical Sciences, Bharatpur, Nepal; ^3^Department of Oral Pathology Awadh Dental College and Hospital Jamshedpur, Jharkhand, India; ^4^Department of Oral Pathology, Sathyabama Dental College and Hospital, Chennai, India; ^5^College of Dental Medicine, Roseman University of Health Sciences, South Jordan, UTAH - 84095, USA; ^6^Centre of Molecular Medicine and Diagnostics (COMManD), Saveetha Dental College and Hospitals, Saveetha Institute of Medical and Technical Sciences, Saveetha University, Chennai, India

## Abstract

Lipoma is one of the benign soft-tissue tumors in the adipose tissue. Oral lipomas comprise 1%–5% of benign oral tumors. They are slow-growing, soft, asymptomatic, solitary tumors. In this case report, a 45-year-old female patient having swelling on the upper right arch for 2 months has been reported. Clinically, the right upper second premolar and first molar were grossly carious, with diffuse swelling on the adjacent vestibule. A provisional diagnosis of the periapical abscess was rendered. Radiological evaluation and routine blood tests were performed. After the tissue was obtained, following teeth extraction and socket curettage, it was sent for histopathological evaluation, and findings suggested intraoral intraosseous lipoma. Clinically, they are asymptomatic, and therefore, if it was not curetted and histopathologic evaluation not done, and only thought to be just a swelling or a periapical abscess, proper treatment could not have been established. This paper will surely bring out the importance of histopathology in routine dentistry and the role of histopathological evaluation of periapical soft tissues.

## 1. Introduction

Lipoma is one of the most common benign soft-tissue neoplasms encountered in the subcutaneous tissue. It is composed of matured adipocytes without any cellular atypia [1]. However, its occurrence within the bone origin associated with the bone is rare. It accounts for less than 0.1% of bone tumors [2]. The most common areas are the calcaneus and the lower long bones. Based on the location of the bone, intraosseous lipoma are of two types intramedullary and intracortical [2]. Approximately, 15%–20% of lipomas are found in the head and neck parts. Among them, 50% occur in the buccal mucosa. The lesion may or may not be encapsulated [3, 4].

Lipoma of bone or intraosseous lipoma is a very rare intraosseous benign neoplasm, formed by adipocytes that can also originate on the surface of the bone [2–4]. It categorizes less than 0.1% of primary bone neoplasms and 15% of them occur on the surface. In the present era, MRI has increased the detection rate of these lesions [2]. They occur usually in the fifth decade of life. Most of the cases occur in the metaphysis of long bones and in the calcaneus. Surface tumors occur over the diaphysis of bones of the extremities [2, 4]. Lipomas are asymptomatic like all other cases [3, 4]. Radiographically, a lucent, well-defined intramedullary lesion, CT, and MRI have features similar to subcutaneous fat. Inside the tumor, mineralized foci may be found [5, 6]. Commonly seen in men, these lesions may develop in all ages but are usually diagnosed around 40 years of age [7]. A maximum of the cases have pain clinically [7]. Histologically, these tumors are composed of mature cells/mature adipocytes, which are slightly larger in size than the nontumorous ones, and may include single spindle cells, focal fat necrosis, calcifications, and bony trabeculae undergoing resorption [2, 7]. Intraosseous lipomas are not based on radiology alone, and therefore, differential diagnosis is very necessary and conducted for a long time [7, 8]. CT scan and Magnetic resonance imaging give a tissue-specific diagnosis [8, 9]. If there is a presence of a predominant fatty component in a lesion, it confirms its benign nature, and hence, no further diagnostic procedures are needed [8–10].

In this case report, a very interesting case is shown which was thought to be a periapical lesion, but after extraction, a gelatinous lesion was attached to the root which was different from a granulation tissue, which urged us to go for histopathological investigation, which was diagnosed as intraosseous lipoma. Therefore, the purpose/aim is to show the role of histopathology and its importance.

## 2. Pathogenesis

The nature of these lipomas is still debatable; the origin could be from medullary adipose tissue, trauma, or reactive changes [3, 10]. The causes are not clear, but still, etiology may be due to endocrine, inflammatory, and mechanical causes. Some cases have shown 12q, 13q, and 6p chromosomal rearrangements [3, 8]. It may also be contributed to the fact that the pathology is due to “hypertrophy theory” where obesity and inadvertent growth of fatty tissue cause intraoral lipoma [11]. There is also another theory stating “metaplasia theory” where these occur due to aberrant differentiation of in situ mesenchymal cells into lipoblast because, from mutable cells of connective tissue, adipose tissue can be derived in any part of the body [10, 11]. Some authors also concluded that lipoma is benign, congenital arising from multipotential cells of the embryo, and remains inactive subclinically, which under hormonal influence develops into adolescence and differentiates into adipose cells [8, 9, 11]. In some cases, trauma and chronic irritation fasten the proliferation of soft tissue and develop a lipoma [9–11].

## 3. Prognosis and Treatment

They are benign lesions, and therefore, their prognosis is very good [4]. The recurrence and relapse of these lesions are rare if curetted properly and excised, malignant transformation has not been so far reported [4]. There are cases of osseous lipoma reported in the maxilla and mandible, but no lesion has so far been reported has occurred with a periapical lesion in the tooth socket [12, 13].

## 4. Case Report

**Figure 1:**
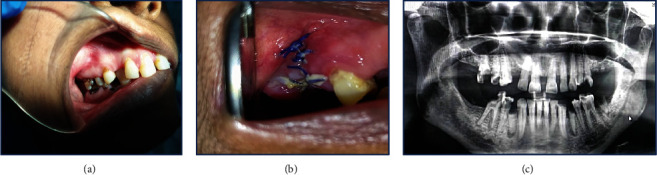
(a) Clinical presentation of the case, diffuse swelling adjacent to the grossly carious teeth on the vestibule, (b) postsurgical healing after 8 days, and (c) the orthopantomography of the patient showing minimal radiolucency on the root apices masked by the maxillary sinus.

A female patient aged 45 walks into the clinic complaining of a slowly growing swelling on the upper right arch since 2 months. The pain was reported to be fluctuating, dull, and intermittent. There was a heaviness and discomfort over that area. On clinical examination, the right upper second premolar and first molar were found to be grossly carious with a diffuse swelling on the vestibule adjacent (Figure 1(a)). The swelling was firm and mild tender on palpation; however, there was no tenderness on those two teeth. An orthopantomogram image was studied, and mild radiolucency was found in the root apices (Figure 1(c)). Ideal apical periodontitis was not appreciated but looking at the clinical presentation and radiography, a provisional diagnosis of the periapical abscess was given. On documentation of the overall scenario extraction of the carious teeth was planned following curettage and follow-up was advised for prosthesis planning in the future.

## 5. Investigations and Treatment/Prognosis of the Case

A routine blood investigation before any surgical procedure such as complete blood count, hemoglobin, blood sugar, bleeding time, and clotting time was done, and it was found to be within normal ranges.

These tests were performed to see any abnormalities in these parameters, if any abnormalities were present then extraction or surgeries may have been delayed or any other options or medications may have been prescribed.

The teeth were then extracted under local anesthesia and on socket curettage, a gelatinous soft mushy tissue was obtained, which was sent for histopathology. A provisional diagnosis of periapical granuloma was rendered.

## 6. Histopathology

**Figure 2:**
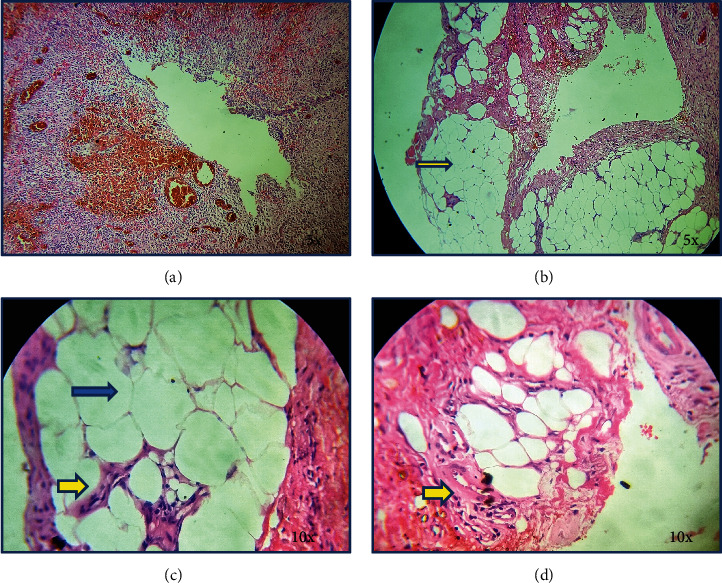
Photomicrograph showing. (a) Stroma showing dispersed chronic inflammation with few areas of degeneration (5× magnification). (b) Mature adipocytes in a lobule separated by thin connective tissue septa (yellow arrow) (5× magnification). (c) Minimal presence of inflammation (yellow arrow), mature adipocytes (blue arrow) (10× magnification). (d) Compressed blood vessels in between the lobules (yellow arrow) (10× magnification).

The histopathology revealed moderate to dense fibrocollagenous stroma with abundant dispersed chronic inflammation with a lot of dilated blood vessels engorged with RBCs. There is also evidence of abundant mature lobules of adipocytes separated by thin fibrous connective tissue septa. The fat cells did not show any atypia and mitotic figures. There was no evidence of a capsule. The inflammatory component was found to be minimal in these areas with few compressed blood vessels (Figure 2). Looking into all the features a diagnosis of lipoma was rendered, since the entire lesion was obtained from the bony socket a confirmed diagnosis of intraosseous lipoma was delivered. The lesion was excised and the healing was uneventful. The postoperative status of the patient after 1 week is shown in Figure 1(b), and follow-up was done every 3 months till 1 year where no recurrence was noted.

## 7. Discussion

Lipoma is a painless soft-tissue tumor of mesenchymal origin, which has slow growth and is usually well-circumscribed. Its origin is in the mature adipose cells [1]. Its frequency is much higher in the head and neck region, but its occurrence is only about (1%–4%) in the oral cavity.

Intraosseous lipoma is rare in occurrence and consists of 0.1% of all bone tumors. They have lots of degenerative changes and shows areas of fat necrosis, cystic changes, and areas of calcification [8].

They are very rare in head and neck bones. They are divided into two subtypes according to the site of origin into intramedullary and intracortical types. Among them, few cases of intramedullary lipoma are reported in the oral region and they are symptomless and diagnosed accidentally on radiographs in patients having come for any other reasons [10].

The cancellous and long tubular bones are the anatomical sites most commonly affected. In long bones, they are found in the metaphysis. Intraosseous lipoma may be traced anywhere in the skeleton. Pain is the main symptom in maximum cases and intraosseous lipomas are found accidentally in imaging studies that are done for other reasons [8].

In some cases, when the tumor becomes large enough results in difficulty in speech and mastication [8, 9]. Due to their yellowish color, and their usual location superficially near the mucosa, intraoral lipomas tend to have an easy diagnosis [14, 15]. They are composed of mesenchymal adipose cells, with a thin mucosa covering, and their occurrence can be in any tissue or organ of the body. They present as solitary tumors. Multiple lesions occur in 5% of the cases. The nature and etiopathogenesis are not very clear and are unknown [8, 9, 14, 15].

They usually grow asymptomatic and slow, which is why patients do not seek treatment [16]. These lesions are not painful, mobile, submucosal nodules, color being yellow when seen clinically. These lesions may be sessile or pedunculated, and have soft to firm consistency [17]. These lesions usually created the problem in mastication and speech as they persist for a longer time.

The gold standard for the proper diagnosis of Lipomas is always based on histopathological evaluation. It is also very necessary to omit malignant lipoma [3, 7, 10, 11].

Classification on the basis of histology is simple lipoma, fibrolipoma, spindle cell lipoma, intramuscular lipoma, chondrolipoma, pleomorphic lipoma, myxoid lipoma, angiolipoma, and sialolipoma [17].

Histopathologically, in intraosseous lipoma, there is a three-stage classification as given by Milgram-stage I-a lesion having mature fat cells without calcification; stage II-a predominantly fat lesion with necrosis and focal calcification/ossifications, and stage III-a lesion having fat having multiple necroses, wide calcifications, cystic degenerations [8].

In other intraoral lipomas, these lesions show thinly encapsulated lesions having mature adipocytes and inconspicuous vascularity [18]. Just like fat, lipomas constitute mature fat cells, but cells vary slightly in size and shape and are somewhat larger, having diameters up to 200 mm [18]. Subcutaneous lipomas have thin encapsulation and have proper lobular patterns [13]. The deep-seated lipomas show irregularity in configuration, majorly based on the localization. These lipomas are usually well vascularized but due to the abundance of the lipocytes, the vascular channels are compressed and appear sparse. Oral lipomas tend to have an occurrence between the sixth and seventh decades of life [3, 12, 13, 18].

The malignant transformation of lipoma is extremely rare in occurrence. Surgical excision is the treatment of choice and shows an excellent prognosis [16, 18]. Difficulty in removal can be seen in some cases in deep-seated lesions [18, 19]. Rarely, recurrence or complications following surgical removal can be seen [19]. Under, Medical management, a steroid injection can be given to cause atrophy of adipose tissue [20]. A good prognosis is seen in lesions less than 2.5 cm in diameter. Injections of a mixture of 1 : 1 parts of lidocaine with triamcinolone acetonide are repeated monthly. Liposuction can also be another treatment option in average (4 to 10 cm) or large-sized (>10 cm) tumors [20, 21]. Surgical excision always remains the treatment of choice for this lesion, among all other nonsurgical treatment regimens [6, 18, 20]. The elimination of differential diagnosis is a must for all practitioners to ensure proper treatment, and surgical excision for the well-being, comfort, and quality of life of the patients. Relapse or recurrence is rare, but long-term follow-up is compulsory [1, 2, 8, 9, 21].

In the present case, it could very well be thought to be a dentoalveolar abscess, sinus inflammation, or any odontogenic lesions, but since it was firm and associated to grossly carious teeth, a provisional diagnosis of periapical abscess was rendered. The key intension to bring forward this case as sometimes the most obvious diagnosis turns out to be different. Lipomas are benign entity, never aggressive but can be a concern for the patient. So, the point which is loud and clear is periapical tissues if obtained attached to the tooth or during curettage must be treated with importance and sent for histopathological evaluation. Any chances of recurrence and the characteristic of the lesion should be well explained to the patient and documented in the case sheet for future usage. So histopathologic evaluation of every case even the smallest, doubtful case is must, and there is a very important role of oral pathologists in this area otherwise the treatment outcome will not be proper.

## 8. Conclusion

Intraoral lipoma is a benign and rare mesenchymal neoplasm, and among them, intraosseous lipoma is considered very rare entity and forms 0.1%.

The most common areas are the calcaneus and the lower long bones. Based on the location of the bone, intraosseous lipomas are of two types intramedullary and intracortical. Approximately, 15%–20% of lipomas are found in the head and neck parts. Among them, 50% occur in the buccal mucosa. The lesion may or may not be encapsulated. Intraosseous lipomas are not based on radiology alone and therefore differential diagnosis is very necessary and conducted for a long time.

Even a simple periapical abscess or granuloma as assessed in the radiograph turned out to be intraosseous lipoma. Clinicians must understand the importance of diagnosis of the oral lesions and also differential diagnosis should be kept in mind for proper understanding and treatment protocols to be taken. Proper curettage, surgical excision must be done with follow-ups after histopathological diagnosis keeping in mind the health of the patient and giving the patient correct direction in their life.

## Data Availability

Research data not shared anywhere.

## References

[1] Agarwal R., Kumar V., Kaushal A., Singh R. K. (2013). Intraoral lipoma: a rare clinical entity. *Case Reports*.

[2] Bhurgri Y., Faridi N., Ayub M., Rehman A. Z. (2000). Lipoma of the bone. *Journal of Pakistan Medical Association*.

[3] Egido-Moreno S., Lozano-Porras A. B., Mishra S., Allegue-Allegue M., Marí-Roig A., López-López J. (2016). Intraoral lipomas: review of literature and report of two clinical cases. *Journal of Clinical and Experimental Dentistry*.

[4] Hoseini A. T., Razavi S. M., Khabazian A. (2010). Lipoma in oral mucosa: two case reports. *Dental Research Journal*.

[5] Morais A. L. G., Mendonça E. F., de Alencar A. H. G., Estrela C. (2011). Intraosseous lipoma in the periapical region of a maxillary third molar. *Journal of Endodontics*.

[6] Cakarer S., Selvi F., Isler S. C., Soluk M., Olgac V., Keskin C. (2009). Intraosseous lipoma of the mandible: a case report and review of the literature. *International Journal of Oral and Maxillofacial Surgery*.

[7] Kaur R., Kler S., Bhullar A. (2011). Intraoral lipoma: report of 3 cases. *Dental Research Journal*.

[8] Kang H. S., Kim T., Oh S., Park S., Chung S. H. (2018). Intraosseous lipoma: 18 years of experience at a single institution. *Clinics in Orthopedic Surgery*.

[9] Sharma P. K., Kundu Z. S., Tiwari V., Digge V. K., Sharma J. (2021). Intraosseous lipoma of the calcaneum. *Cureus*.

[10] Basheer S., Abraham J., Shameena P. M., Balan A. (2013). Intraosseous lipoma of mandible presenting as a swelling. *Journal of Oral and Maxillofacial Pathology*.

[11] Campbell R. S. D., Grainger A. J., Mangham D. C., Beggs I., Teh J., Davies A. M. (2003). Intraosseous lipoma: report of 35 new cases and a review of the literature. *Skeletal Radiology*.

[12] Furlong M. A., Fanburg-Smith J. C., Childers E. L. (2004). Lipoma of the oral and maxillofacial region: site and subclassification of 125 cases. *Oral Surgery, Oral Medicine, Oral Pathology, Oral Radiology, and Endodontology*.

[13] Dehghani N., Razmara F., Padeganeh T., Mahmoudi X. (2019). Oral lipoma: case report and review of literature. *Clinical Case Reports*.

[14] Atarbashi-Moghadam S., Lotfi A., Mehdizadeh M., Atarbashi-Moghadam F. (2021). Clinicoradiographic features and histopathologic variations of intraosseous lipoma: report of a case and review of the literature. *Case Reports in Dentistry*.

[15] Kumar L. K., Kurien N. M., Raghavan V. B., Menon P. V., Khalam S. A. (2014). Intraoral lipoma: a case report. *Case Reports in Medicine*.

[16] Alharbi A. S. (2020). Intraoral lipoma of the cheek–a case report with a one-year follow-up and review of literature. *Cureus*.

[17] Naik P., Samatha Y., Kumar V., Kumar K. (2013). Intraoral lipoma: a rare case report and review of literature. *Journal of Clinical and Diagnostic Research*.

[18] Filho G. A. N., Caputo B. V., dos Santos C. C. (2010). Diagnosis and treatment of intraoral lipoma: a case report. *Journal of the Health Sciences Institute*.

[19] Palczewski P., Świątkowski J., Gołębiowski M., Błasińska-Przerwa K. (2011). Intraosseous lipomas: a report of six cases and a review of literature. *Polish Journal of Radiology*.

[20] Sanjuan A., Dean A., Garcia B., Alamillos F., Roldan E., Blanco A. (2017). Condylar intramedullary intraosseous lipoma: contribution of a new case and review of the literature. *Journal of Clinical and Experimental Dentistry*.

[21] Maksoud C., Aoun G. (2022). Intraosseous lipoma: a report of a case in the mandibular symphysis region. *Cureus*.

